# pH Driving the Self-Assembly of Hydrolyzed Edible Dock Protein and Myricetin

**DOI:** 10.3390/foods15111879

**Published:** 2026-05-26

**Authors:** Xiulan Wang, Atif Arshad, Jin Liang

**Affiliations:** Key Laboratory of Jianghuai Agricultural Product Fine Processing and Resource Utilization, Ministry of Agriculture and Rural Affairs, Anhui Engineering Research Center for High Value Utilization of Characteristic Agricultural Products, College of Food and Nutrition, Anhui Agricultural University, Hefei 230036, Chinaatifarshad0000@gmail.com (A.A.)

**Keywords:** edible dock protein, myricetin, self-assembly, interaction

## Abstract

The amphiphilic peptides formed by moderate enzymatic hydrolysis of proteins can self-assemble into various structures at different pH levels, resulting in differences in the encapsulation efficiency of hydrophobic substances. In this work, a new plant protein of edible dock protein (EDP) was moderately hydrolyzed by *Bacillus licheniformis* proteinase to prepare hydrolyzed edible dock protein (HEDP). The self-assembly behavior and interaction mechanism of HEDP with myricetin (Myr) at different pHs were explored. The results showed that the loading capacity of Myr by HEDP was 13.86% higher than that of EDP before enzymatic hydrolysis. Moreover, under pH 9.0, the zeta potential, particle size, and PDI of the Myr-HEDP were −34.77 mV, 119 nm, and 0.33, respectively. Meanwhile, the Myr at this pH had the highest encapsulation efficiency (94.55%) and loading capacity (24.8%). Transmission electron microscopy exhibited that the Myr-HEDP nanomicelles had an obvious core–shell structure. Spectroscopy experiments confirmed that there were varying intensities of hydrogen bonding and hydrophobic interactions between HEDP and Myr at different pHs, wherein the binding intensity was largest at pH 9.0. Additionally, the stability evaluation indicated that the UV, thermal, storage, and digestive stability of Myr within the Myr-HEDP nanomicelles at pH 9.0 were more stable than at other pH conditions. In summary, pH 9.0 was more conducive to the self-assembly of HEDP and Myr, forming stable composite nanomicelles. This study will provide an important input into designing more stable and efficacious EDP delivery systems.

## 1. Introduction

Myricetin (Myr) is a flavonoid compound that can protect the cardiovascular and cerebrovascular systems and prevent chronic diseases, such as atherosclerosis, cancers, and diabetes [1,2]. However, myricetin has poor water solubility and low environmental stability, which limits its effective utilization in functional foods and drugs [3,4,5]. To enhance the stability of myricetin, various protein carriers have been developed recently. For instance, Chen et al. have designed a shrimp ferritin dual-compartmental micro-droplet nanocage for loading myricetin, giving the encapsulation efficiency and loading capacity of 28.36% and 2.33%, respectively [6]. Moreover, this delivery system enhanced the stability and anti-glioblastoma cell activity of myricetin. Guo et al. have developed casein nanomicelles for encapsulating myricetin with EE and LC of 81.20% and 9.00%, respectively [7]. Rat perfusion experiments indicated that the small intestine absorption rate and bioavailability of the encapsulated myricetin were higher than those of free myricetin. These research studies suggested that a protein delivery system is an effective strategy to improve the nutritional value and health benefits of myricetin. Nevertheless, the loading capacity and controlled release characteristics are still unsatisfactory.

Edible dock, belonging to the *Polygonaceae* family, is a new food raw material [8]. In recent years, increasing attention has been garnered for edible dock because of its high content of proteins (30~45%). Moreover, the hydrophobic amino acid content of edible dock protein is more than 45%, which can form a hydrophobic cavity for the encapsulation of hydrophobic small molecules via self-assembly. Enzymatic hydrolysis is often used to upgrade the structural and functional characteristics of proteins, such as spatial conformation, water solubility, gelation, etc. [9,10]. In fact, the partially hydrolytic proteins can form amphiphilic peptide chains and further self-assembly into a core–shell structure, including micelles, nanotubes, and nanoparticles [4,11,12]. These nanostructures can be used as a delivery system for the encapsulation of bioactive chemicals to enhance their stability and intestinal absorption. Compared with conventional protein-based carriers, hydrolyzed edible dock protein possesses unique advantages due to its plant-based origin, high hydrophobic amino acid content, and enhanced self-assembly capability after enzymatic hydrolysis. These characteristics may contribute to improved encapsulation performance and nanomicelle stability for hydrophobic bioactive compounds.

It has been reported that pH is an important determinant that can affect the self-assembly behavior and interaction strength between protein molecules and bioactive compounds by regulating the spatial structure, charge distribution, and solubility of proteins. This made it possible for the pH-induced approach to drive the self-assembly of protein and small molecules in a simple, efficient, and economical strategy. For instance, Ferritin’s ability to dissociate and recombine at varying pH levels has been studied by Zhou et al. [13]. The results showed that the ferritin nanoshells can split into individual subunits at pH 2.0 or 11.0 and then reconstitute into a cage-like structure at a pH of 7.5. And the proanthocyanidins were effectively encapsulated in the protein’s inner cavity during reconstitution, which improved the substance’s water solubility, light stability, and cellular absorption activity. Wang et al. have constructed a nano-biomaterial with tunable mesoscopic structures [14]. This can be achieved by dissolving casein micelles and rice proteins under pH conditions of 12.0 and slow neutralization at pH 7.0 for self-assembly. Moreover, the formed protein composites were used as a delivery system for loading apigenin to improve its anti-tumor effect. Dai et al. reported that curcumin can be successfully incorporated into casein carriers by a pH-driven approach [15]. However, the combination of partial enzymatic hydrolysis and pH-driven methods in the construction of edible dock protein delivery systems has not been reported yet.

In the present work, the *Bacillus licheniformis* enzyme was used to partially hydrolyze edible dock protein for obtaining amphiphilic peptides. On this basis, the partially hydrolyzed edible dock protein peptides and hydrophobic myricetin were self-assembled under different pH conditions. The self-assembly behavior and interaction mechanism of hydrolyzed edible dock protein and myricetin were investigated. The results will offer scientific guidance for efficient development of delivery systems and deeper utilization of new plant proteins.

## 2. Materials and Methods

### 2.1. Materials

The supplier of myricetin (Myr, ≥98%) was ChemFaces Co., Ltd. (Wuhan, China). Harbin Sancao Agricultural Technology Co., Ltd. (Harbin, China) provided the edible dock. The *Bacillus licheniformis* enzyme (≥2.4 U/g, product No. P4860) was purchased from Sigma Company (St. Louis, MO, USA). Every reagent was of analytical grade.

### 2.2. Preparation of the Myr-Loaded Hydrolyzed Edible Dock Protein Nanomicelles Under Different pH Conditions

Initially, 30 mL of phosphate buffer solution was filled with edible dock proteins (EDPs, 180 mg), which were then swirled for four hours at room temperature. Upon removing insoluble materials through centrifugation at 4000 rpm for 10 min, the supernatant was extracted and used as the EDP stock solution [8]. To obtain the hydrolyzed edible dock protein (HEDP) nanomicelles, a certain amount of *Bacillus licheniformis* enzyme (0.12 mL) was added to 20 mL of EDP stock solution and incubated at 50°C for 30 min while maintaining the pH at 7.0, followed by inactivating the enzyme in a 100°C water bath for 10 min. The pH of the HEDP dispersion was then adjusted dropwise to 3.0, 5.0, 7.0, 9.0, and 11.0 using 1 M HCl or 1 M NaOH under continuous magnetic stirring until stabilization. These pH conditions were selected to systematically represent strongly acidic, acidic, neutral, alkaline, and strongly alkaline environments for investigating the self-assembly behavior and stability characteristics of the HEDP-Myr nanomicellar system under different pH conditions. The preparation of the Myr stock solution (10 mg/mL) required the dissolution of myricetin in anhydrous ethanol. In order to encapsulate Myr, the Myr stock solution (1 mL) was slowly injected into the HEDP nanomicelles (25 mL) under different pHs (3.0, 5.0, 7.0, 9.0, and 11.0). The resulting mixed solutions were continuously stirred for 30 min under dark conditions to facilitate ethanol volatilization and then centrifuged (8000 rpm, 20 min) to remove the unembedded myricetin. Myr-loaded hydrolyzed edible dock protein nanomicelles comprised the supernatant.

### 2.3. Encapsulation Efficiency (EE) and Loading Capacity (LC)

The composite nanomicelles were mixed with anhydrous ethanol at a volume ratio of 1:9 and sonicated for 30 min at 25 °C in order to extract myricetin from the Myr-HEDP nanomicelles. To extract the supernatant, the mixed systems were centrifuged for 5 min at 3000 rpm. The absorbance of Myr in the supernatant was determined at 385 nm by an enzyme labeling instrument (Pontyclun, UK). Subsequently, using the expressions (1–3), where X and Y represent the concentration of Myr (µg/mL) and optical density value, respectively, the EE and LC of Myr were determined.

(1) *Y* = 0.029 *X* + 0.0794, R^2^ = 0.9985

(2) $$EE=\frac{encapsulated\;flavonoids}{total\;flavonoids}\times 100$$

(3) $$LC=\frac{total\;flavonoids-free\;flavonoids}{total\;flavonoids}\times 100$$

### 2.4. Characterization of the Myr-HEDP Nanomicelles

A dynamic light scattering laser particle analyzer (ZSU3100, Malvern, UK) was used to assess the polydispersity index (PDI), zeta potential, and particle size of HEDP and Myr-HEDP at 25°C. To determine the surface hydrophobicity of the HEDP nanomicelles, recrystallized pyrene was used as a fluorescent probe. A fluorescence spectrometer (FL 6500, PerkinElmer, Shelton, CT, USA) was used to record the samples’ fluorescence spectra at 340–400 nm following the 48 h reaction between pyrene and HEDP solution at 25 °C. For fluorescence measurements, the HEDP samples were diluted 10-fold prior to analysis, and the final concentration of HEDP was set at 0.6 mg/mL. In the thermodynamic analysis, the HEDP concentration was fixed at 0.6 mg/mL, and myricetin concentrations were set to 20–60 μmol/L (20, 30, 40, 50, 60 μmol/L). Using a transmission electron microscope (TEM, HT-7700, Tokyo, Japan), the micromorphology of the Myr-HEDP was examined.

### 2.5. Contact Angle and Surface Tension

The surface tension and contact angle of the nanomicelle systems were measured using a contact angle meter (SDC-200S, Dongguan, China). The contact angle was recorded when 1 μL of the droplet fell onto the sample stage. The surface tension was determined with a time interval of 0.5 s using a hanging drop method.

### 2.6. Fourier Transform Infrared (FT-IR)

The secondary structures, functional groups of self-assembled HEDP, Myr-HDEP, and the intermolecular forces between HEDP and Myr were investigated by an FT-IR spectrometer (NicoletTMiS™ 50, Thermo Scientific, Waltham, MA, USA) at different pH conditions. In detail, the dried KBr (100 mg) and 1 mg of lyophilized sample were combined, then pressed into tablets that were scanned between 4000 and 400 cm^−1^ wavelengths.

### 2.7. Thermal, UV, and Storage Stability

Samples were taken at specific intervals after the Myr and the Myr-HEDP were heated to 60 °C for 120 h or exposed to 254 nm UV light for 120 h at room temperature in order to examine the thermal and UV stability of Myr in Myr-HEDP. Then, the contents of Myr were determined by an enzyme labeling instrument, where A_0_ is the Myr content of the initial sample before heating or UV treatment, and A_t_ is the Myr content of the sample after addition or UV treatment. The Myr-HEDP nanomicelles were kept at 4 °C for three months in order to assess their storage stability. The nanomicelles’ particle size, PDI, and zeta potential were also measured, as well as the residual amount of Myr.

(4) Retention rate = A_t_/A_0_ × 100%

### 2.8. Fluorescence Spectroscopy

A FL 6500 fluorescence spectrometer (PerkinElmer, USA) was used to measure the fluorescence spectra of HEDP and Myr-HEDP. The concentrations of the nanomicelles were suitably adjusted; the excitation, emission wavelengths, and emission slit width were set to 295 nm, 300–500 nm, and 10 nm, respectively. The scanning rate was tuned at 1000 nm/min. HEDP and Myr-HEDP’s three-dimensional fluorescence spectra were obtained between 200 and 500 nm for emission and between 200 and 400 nm for excitation. A constant test temperature of 25 °C was used.

### 2.9. Molecular Fluorescence Analysis

The Myr-HEDP nanomicelles prepared under different conditions (pH 7.0, 9.0, and 11.0) were incubated at the three conditions (25 °C, 35 °C, and 45 °C) for 1 h, respectively. The fluorescence emission intensity of each nanomicelle was measured by a fluorescence spectrometer. The excitation wavelength of the fluorescence spectrum was 295 nm, the emission wavelength ranged from 300 to 450 nm, and the slit width for both excitation and emission was 2.5 nm. The Stern–Volmer equation was used to examine the fluorescence quenching mechanism [16].

(5) *F*_0_/*F* = 1 + *K_q_*τ_0_[*Q*] = 1 + *K_SV_*[*Q*]

where *F*_0_ represents HEDP’s maximum fluorescence intensity, and *F* represents Myr-HEDP’s highest fluorescence intensity. The Stern–Volmer quenching constant is *K_SV_*, the concentration of Myr is [Q], the fluorescence quenching rate constant is *K_q_*, and τ_0_ is 10^−8^ s. The following formula was used to determine Myr-HEDP’s binding site (n) and binding constant (*K_a_*):

(6) lg[(*F*_0_ − *F)*/*F*] = lg*K_a_* + *n*lg[*Q*]

Van ‘t Hoff equations are used to examine the enthalpy change (*ΔH*), entropy change (*ΔS*), and Gibbs free energy (*ΔG*):

(7) ln *K_a_* = −*∆H*/(R*T*) + *∆S*/R

(8) *∆G* = *∆H* − *T∆S*

In this case, R is the gas constant [8.314 J·(mol·K)^−1^], T is the test temperature, and *K_a_* is the binding constant.

### 2.10. In Vitro Release Characteristics of Myr

For the purpose of studying the Myr-HEDP digestive characteristics in vitro, simulated saliva fluid (SSF), simulated gastric fluid (SGF), and simulated intestinal fluid (SIF) were prepared. In simulated saliva fluid (SSF, pH = 7.0), the concentration of CaCl_2_ was 11.1 mg/mL, and the activity of α-amylase was 75 U/mL. In simulated gastric fluid (SGF, pH = 1.5), the concentration of NaCl and the activity of pepsin were set at 2 mg/mL and 50 U/mL, respectively. In simulated intestinal fluid (SIF, pH = 7.2), the concentration of KH_2_PO_4_ was 6.8 mg/mL, and the activities of trypsin and pancreatic amylase were 40 U/mL and 75 U/mL, respectively. First, 15 mL of Myr-HEDP nanomicelles or free Myr solution was added to an equal volume of SSF. The mixture was then shaken for two minutes at 37 °C on a shaking table. After that, 15 mL of the aforementioned mixture was combined and mixed with 15 mL of SGF, and the shaking table was allowed to incubate for 60 min at 37 °C. Finally, the mixed liquid was mixed with SIF in a 1:1 ratio and incubated at the same temperature for 120 min. Samples with different digestion times were collected, and the residual amount of Myr, as well as the PDI and zeta potential of Myr-HEDP, were determined as described in Section 2.3 and Section 2.4. The present study mainly focused on the stability and release behavior of Myr-HEDP nanomicelles during simulated gastrointestinal digestion. The bioaccessibility calculation and intestinal absorption behavior were not evaluated in this study and require further investigation in future research.

### 2.11. Statistical Analysis

Three replications were conducted in each experiment, and the data were presented as mean ± standard deviation. The data visualization was done using Origin 2021 software. Statistical analysis was conducted using SPSS 19.0 software. One-way analysis of variance (ANOVA) followed by Tukey’s HSD test was used to determine significant differences among the means at *p* < 0.05. Normality and homogeneity of variance assumptions were verified prior to analysis. Different lowercase letters in the figures indicate significant differences among groups at *p* < 0.05.

## 3. Results and Discussion

### 3.1. Characterization of Myr-HEDP Composite Nanomicelles at Different pHs

**Figure 1:**
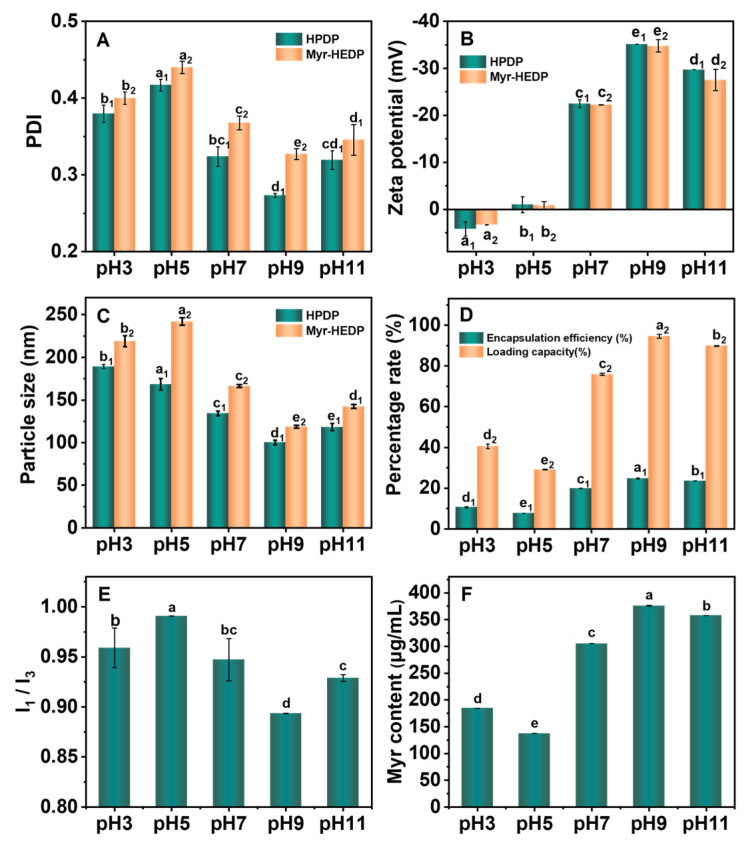
Characterization of Myr-HEDP composite micelles at different pH values: (**A**) PDI; (**B**) zeta potential; (**C**) particle size; (**D**) encapsulation efficiency and loading capacity of Myr in self-assembled HEDP nanomicelles; (**E**) surface hydrophobicity; (**F**) solubility of Myr. Different letters above the bars indicate statistically significant differences among samples (*p* < 0.05). Bars sharing the same letter are not significantly different.

The zeta potential, particle size, and PDI values of HEDP and Myr-HEDP nanomicelles were examined in order to investigate the effect of pH values on the physicochemical properties of nanocarriers. According to Figure 1A,C, at various pH levels, the PDI and particle size of HEDP were as follows: pH 5.0 > pH 3.0 > pH 7.0 > pH 11.0 > pH 9.0, whereas the zeta potential’s absolute value displayed the opposite trend. This phenomenon showed that while at pH 9.0, the particle size and PDI of HEDP nanomicelles were the smallest, and the zeta potential was the largest, that is, the stability was the best under this condition. This was because pH 9.0 was far from the isoelectric point of HEDP (pI 4.0). At this time, the electrostatic repulsion between proteins increased, which promoted dissociation and reorganization of HEDP and hydrogen bonding with water around the protein as well as ionic bonding with salts in solution, resulting in enhanced hydrophilicity of the outer end and hydrophobicity of the inner end of the binding site [17]. However, when the pH was increased to 11.0, the protein unfolded faster than the assembly rate, resulting in excessive dissociation and decreased stability of the nanomicelles. As Figure 1B illustrates, the zeta potential of HEDP and Myr-HEDP nanomicelles shifted from negative to positive at pH < 5.0, which might be attributed to the enhanced protonation of the acidic amino acid side chain at pH < pI, which caused the zeta potential to become positive at pH values below 5.0. In addition, there was only a small difference in the zeta potential values of HEDP nanomicelles before and after loading Myr, indicating that the interaction between HEDP and Myr was less dependent on electrostatic interactions.

Figure 1D showed the encapsulation efficiency and loading capacity of HEDP to Myr at different pH values. It was observed that HEDP had a good encapsulation effect on Myr, especially at pH 9.0, and the best encapsulation effect of HEDP on Myr was achieved (94.55% loading rate and 24.78% loading amount). It is worth noting that the loading rate of HEDP to Myr has increased by 13.86% compared to EDP [18]. Combined with the analysis of the previous results of PDI and zeta potential, the increase in pH made the nanomicelles a more stable system and formed a larger hydrophobic cavity. In order to load myricetin, Chen et al. designed a shrimp ferritin dual-compartmental micro-droplet nanocage, yielding EE and LC values of 28.36% and 2.33%, respectively [19]. Guo et al. have developed casein nanomicelles for encapsulating myricetin, with EE and LC of 81.20% and 9.00% [7]. Comparatively speaking, it is discovered that HEDP nanomicelles have a better loading capacity on Myr than other previously documented delivery systems. According to the aforementioned findings, HEDP nanomicelles might be an effective Myr delivery method. Overall, the HEDP nanomicellar system exhibited significantly superior encapsulation efficiency and loading capacity compared with previously reported myricetin delivery systems. The enhanced loading performance may be attributed to the improved self-assembly behavior, stronger hydrophobic interactions, and greater structural stability of HEDP nanomicelles under alkaline conditions. These findings indicate that HEDP nanomicelles possess considerable potential as an efficient plant protein-based delivery system for hydrophobic bioactive compounds.

Surface hydrophobicity (Ho) reflects the quantity of hydrophobic amino acid residues exposed on the protein surface [20]. The surface hydrophobicity of HEDP reduced when the pH rose from 5.0 to 9.0, as Figure 1E illustrates. The reason might be an increase in the net negative charge of the protein, which decreased the proportion of hydrophobic regions on the surface of HEDP [21]. In addition, the I_1_/I_3_ ratio of HEDP was the lowest when pH was 9.0, indicating that HEDP with pH 9.0 has the lowest surface hydrophobicity, which is advantageous for the stability of the nanomicelle system, as well as for the encapsulation of hydrophobic substances. This result was in line with the previously mentioned findings.

The poor aqueous solubility of Myr was a major challenge, limiting its effective bioavailability. As shown in Figure 1F, the solubility of free Myr in solution was just 0.24 μg/mL. The solubility of Myr increased significantly after the addition of HEDP, especially in HEDP at pH 9.0, where Myr solubility reached 376 μg/mL. This was attributed to the fact that HEDP possessed a large hydrophobic cavity, which allowed hydrophobic Myr to enter its hydrophobic cavity, thus effectively increasing the water solubility of Myr. Notably, the significant increase in Myr solubility pH change and partial enzymatic digestion of proteins could increase the possibility of further use of Myr in food and pharmaceuticals.

### 3.2. Microscopic Morphology

**Figure 2:**
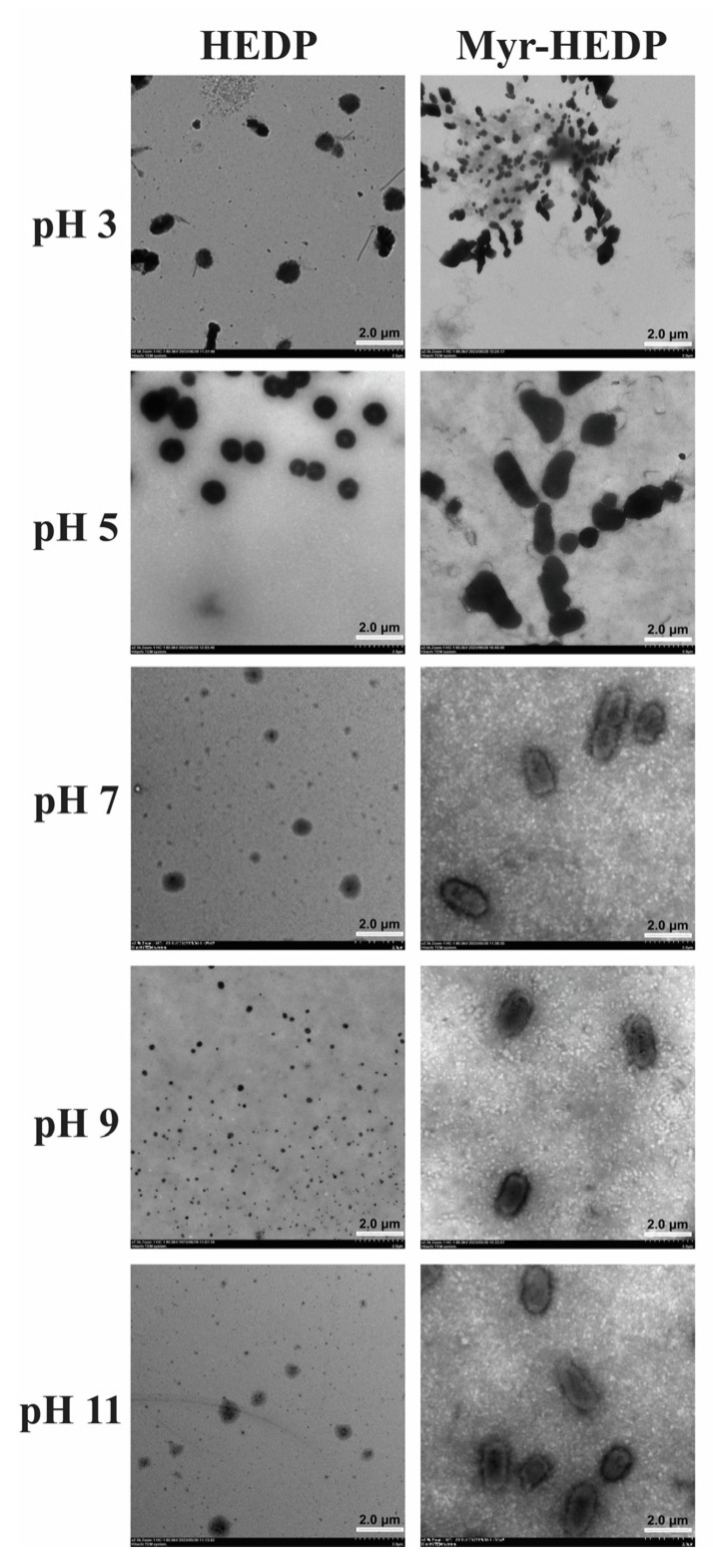
TEM of Myr-HEDP composite micelles at different pH values.

The microstructure of HEDP and Myr-HEDP composite systems treated under five pH conditions was analyzed by TEM. Figure 2 shows the TEM image at 2 μm. At the same magnification, HEDP treated with pH 9.0 has the most uniform particle size and consistent distribution. When HEDP was treated at pH 3.0 and 5.0, the particle size increased, which might be caused by a decrease in electrostatic repulsion between particles near the HEDP isoelectric point (pI 4.0), which led to this aggregation trend. When Myr was added to HEDP with different pH values, the form of Myr-HEDP was also different. When pH was 3.0 and 5.0, Myr-HEDP had a loose structure, an irregular surface, and uneven dispersion, which may be due to the complexation between particles. When pH was 7.0, 9.0, and 11.0, Myr-HEDP had a smooth surface, compact structure, small size, and obvious putamen structure. This was in agreement with the above particle size and PDI results.

### 3.3. Interface Characteristic Analysis

**Figure 3:**
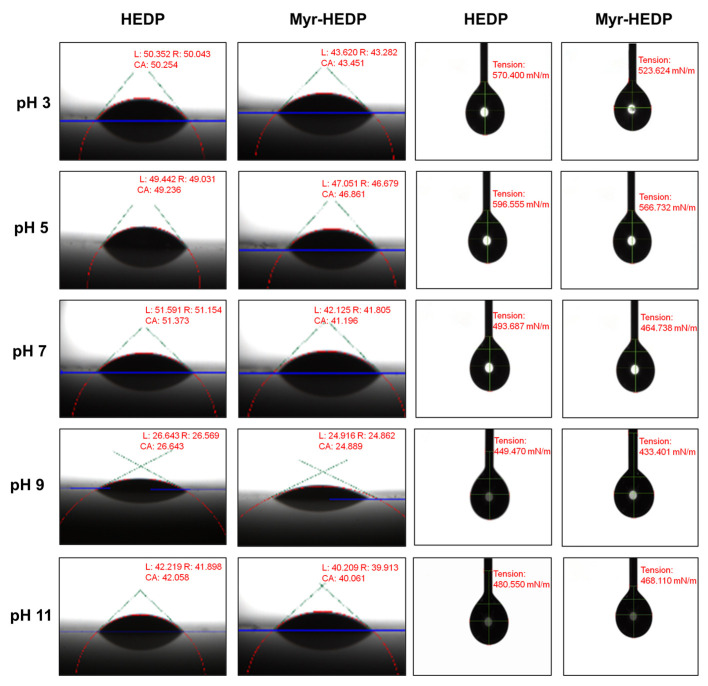
Contact angle and surface tension of Myr-HEDP composite micelles at different pH values.

The interface properties of HEDP and Myr-HEDP nanomicelles were investigated by surface tension and contact angle (Figure 3). According to the findings, the contact angle and surface tension of HEDP and Myr-HEDP under alkaline conditions are smaller than those under acidic conditions. The smaller contact angle and surface tension indicated higher hydrophilicity, which was more favorable for nanomicelle formation [22]. Furthermore, under alkaline conditions, the protein structure unfolds and reorganizes, and the hydrophobic groups are more readily exposed and reduce the interfacial tension, resulting in increased amphiphilicity and interfacial activity of the nanomicelles. Among these composite nanomicelles, Myr-HEDP had the lowest surface tension and contact angle at pH 9.0. Moreover, at all pH conditions, HEDP exhibited a decrease in both contact angle and surface tension when bound to Myr, which could be attributed to the fact that Myr entered the hydrophobic cavity of HEDP and interacted with the hydrophobic sites exposed by HEDP, and these interactions introduce hydrophilic groups (e.g., carboxyl, hydroxyl, etc.) into HEDP to mask its hydrophobic clusters, which results in a decrease in the hydrophobicity of HEDP [6]. In addition, the reaction between a polyphenol such as populin and proteins alters the secondary and tertiary structure of proteins, affecting their surface properties and making them more hydrophilic [23]. Similar results were obtained for the interactions between curcumin and glutaminase-modified SPI [24] and tannic acid and sodium caseinate [25].

### 3.4. Fluorescence Spectroscopy

**Figure 4:**
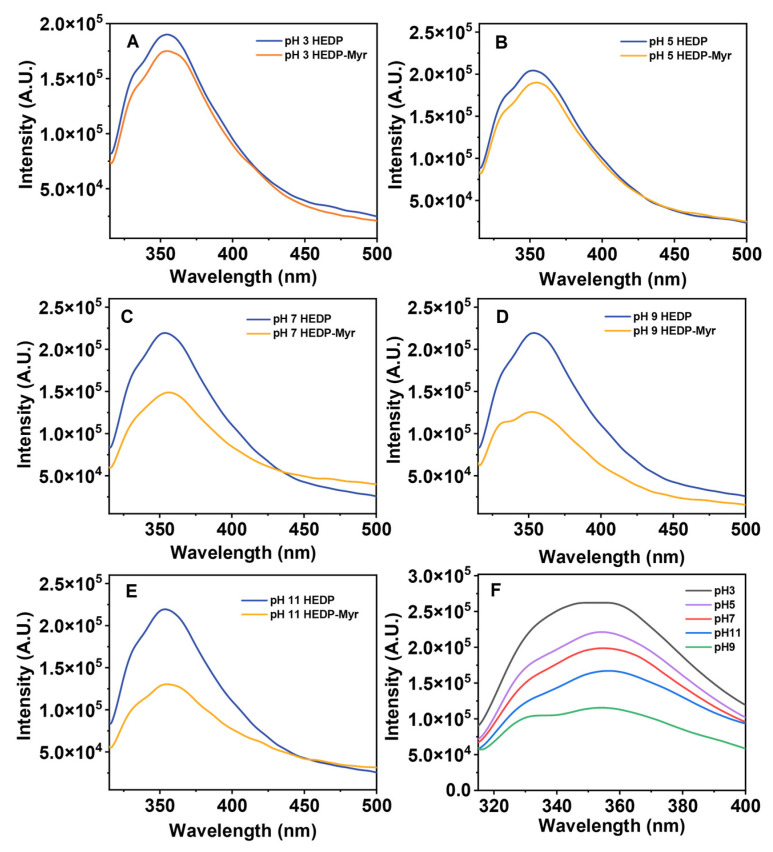
Fluorescence bursts: (**A**) pH 3.0; (**B**) pH 5.0; (**C**) pH 7.0; (**D**) pH 9.0; (**E**) pH 11.0; (**F**) HEDP-Myr at different pH values.

Fluorescence spectroscopy can provide information about protein conformation, dynamics, and the ability of proteins to bind and interact with hydrophobic substances [26,27]. As can be seen from Figure 4A–E, the addition of Myr caused obvious fluorescence quenching and a redshift of HEDP, in which the degree of fluorescence quenching was pH 9.0 > pH 11.0 > pH 7.0 > pH 3.0 > pH 5.0 in descending order. This might be due to the strongest binding ability of HEDP to Myr at pH 9.0. In addition, the fluorescence intensity for HEDP was different for different pH conditions, indicating that the surface proteins were in different microenvironments. Zhu et al. examined the fluorescence spectra of apigenin and β-lactoglobulin at different pH values [28]. They found that the proteins’ fluorescence intensities under alkaline conditions were also shown to be enhanced relative to acidic conditions, and they deduced that the protein’s Tanford transition may be the cause of this rise in fluorescence intensity. During this process, some of the fluorescent group residues’ solvent accessibility was also improved, and these residues became more exposed, leading to an increase in the fluorescence intensity [29].

**Figure 5:**
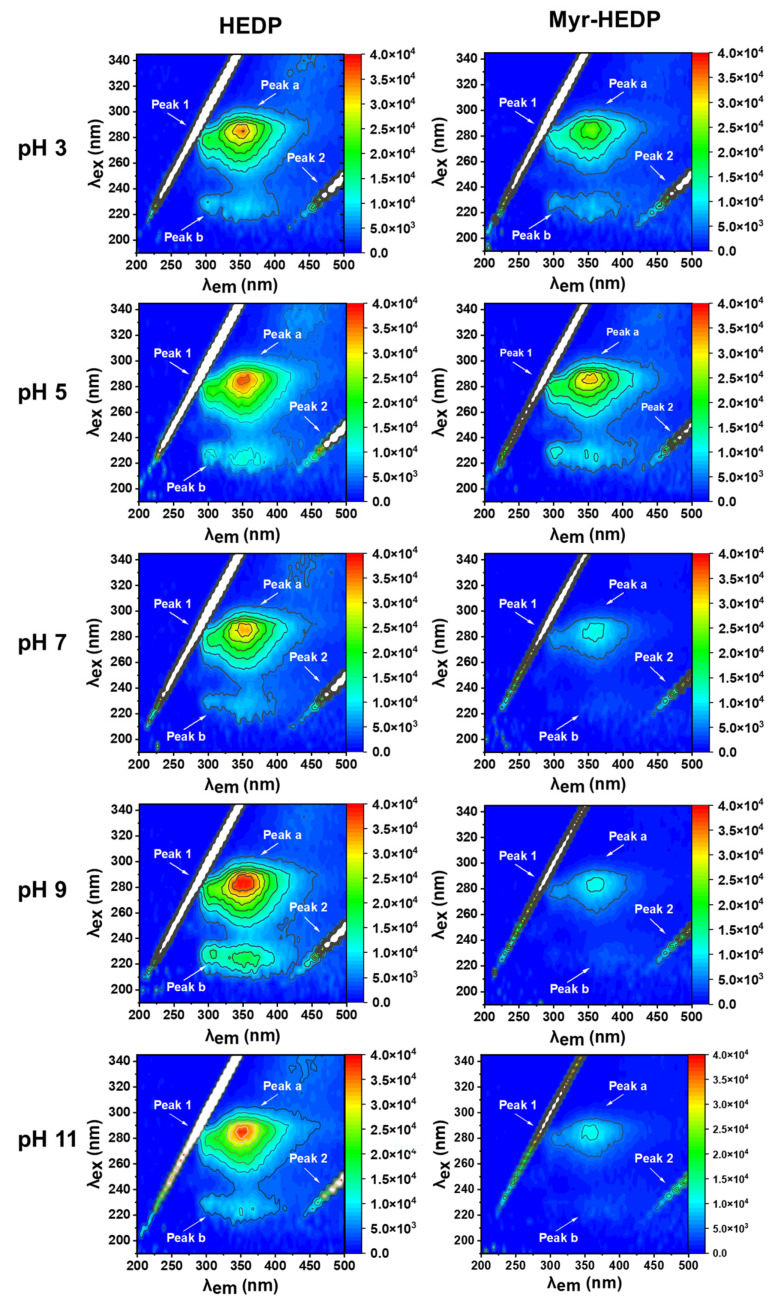
Three-dimensional fluorescence spectra of Myr-HEDP composite micelles at different pHs.

Three-dimensional fluorescence spectroscopy is a useful spectroscopic method to probe the conformational changes of proteins. Figure 5 shows the three-dimensional fluorescence spectra of HEDP and Myr-HEDP under different pH conditions. Peak a (λex = 280 nm) mainly reflects the spectral characteristics of Trp and Tyr residues. Due to the binding of HEDP to Myr, the spatial conformation of HEDP changed, and ultimately, the fluorescence intensity of all the proteins was reduced. The fluorescence quenching order was in line with Figure 4A–F, indicating that pH 9.0 produced the highest degree of fluorescence quenching. In addition, a notable drop in the fluorescence intensity of peak b (λex = 220 nm) occurred after the addition of Myr. It was reported that the peptide backbone lengthening generated by Myr was the primary source of the fluorescence quenching of peak b.

### 3.5. Quenching Mechanism

**Figure 6:**
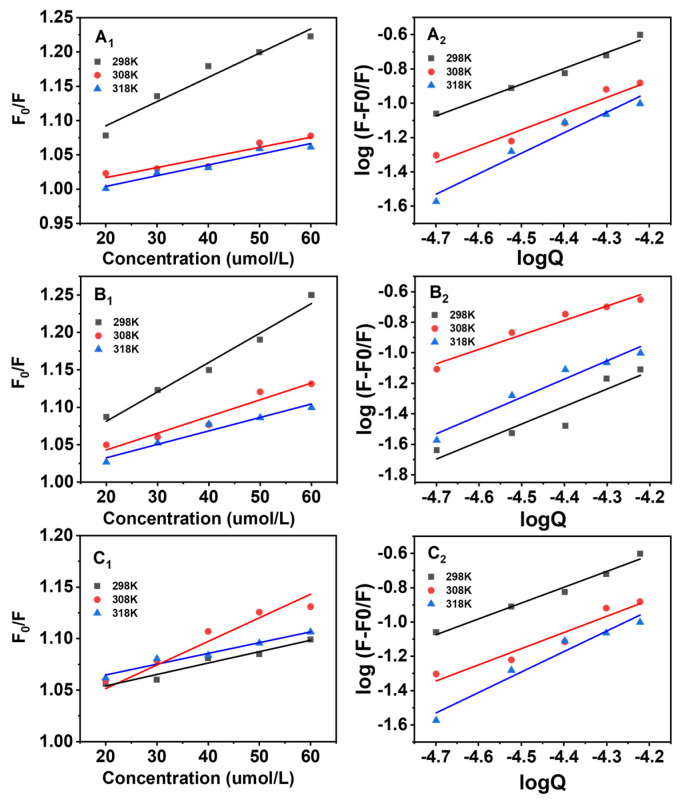
Stern–Volmer curves of flavonoid compounds bound with HEDP at different temperatures. (**A_1_**,**A_2_**) pH 7.0; (**B_1_**,**B_2_**) pH 9.0; (**C_1_,C_2_**) pH 11.0.

Figure 6 and Table 1 showed the interaction mechanisms of Myr-HEDP at pH 7.0, pH 9.0, and pH 11.0, and the *K_SV_* value decreases with increasing temperature, indicating that static quenching is the mechanism of Myr-induced fluorescence quenching of HEDP [30]. Importantly, the *Ka* values of HEDP at pH 9.0 were higher than those at pH 7.0 and pH 11.0, indicating that the binding affinity of HEDP to Myr was the highest at pH 9.0. Enthalpy (*ΔH*), entropy (*ΔS*), and free energy change (*ΔG*) were calculated to determine how HEDP and Myr interact. Table 1 shows that at all pH settings, *ΔG* was negative, suggesting that the binding process between HEDP and Myr was spontaneous. Additionally, the signs of entropy change (*ΔS*) and enthalpy change (*ΔH*) can be used to establish the type of interaction force [31]. Table 1 shows that at all pH conditions (7.0, 9.0, and 11.0), *ΔH* and *ΔS* were less than 0, indicating that hydrogen bonding and van der Waals forces were the main binding forces for the formation of Myr-HEDP complexes. And because sites of charged amino acid residues on HEDP molecules were more likely to interact with Myr to form hydrogen bonds, Myr molecules may be more inclined to bind here.

### 3.6. FT-IR Spectroscopy

**Figure 7:**
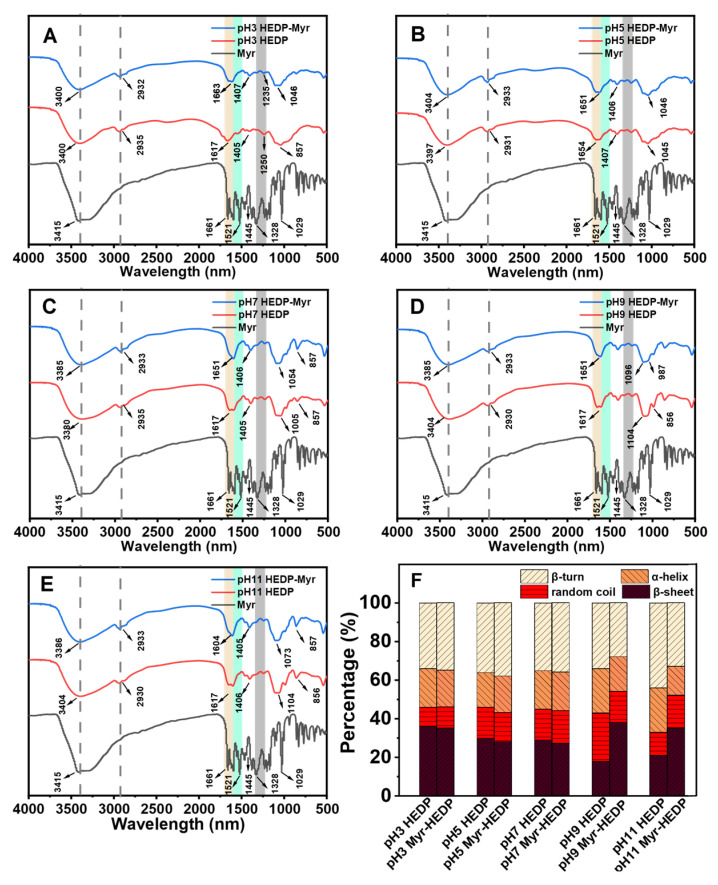
FT-IR curves of HEDP, Myr-HEDP: (**A**) pH 3.0; (**B**) pH 5.0; (**C**) pH 7.0; (**D**) pH 9.0; (**E**) pH 11.0; (**F**) secondary structures of HEDP and Myr-HEDP at different pH values.

The structural changes induced by the self-assembly of HEDP and the intermolecular interactions between HEDP and Myr under different pH environments were analyzed by FT-IR spectroscopy. As shown in Figure 7A–E, the locations of the characteristic peaks of HEDP at different pH values were also different. For example, when pH was 9.0, HEDP characteristic peaks appeared at 3404 cm^−1^, 2930 cm^−1^, 1617 cm^−1^, 1104 cm^−1^, and 856 cm^−1^, while at other pHs, HEDP characteristic peaks changed. In addition, the FT-IR spectra of Myr showed some characteristic peaks, such as the carbonyl peak at 1671 cm^−1^ and the benzene ring skeleton at 1445 cm^−1^ and 1601 cm^−1^. However, the spectra of Myr-HEDP and HEDP almost overlapped. These results indicated that Myr successfully entered the hydrophobic region of HEDP and masked the characteristic peaks of Myr. Besides, there were no new peaks in the spectra of Myr-HEDP compared to HEDP, suggesting that there was a predominantly non-covalent interaction between HEDP and Myr. However, when Myr bound to HEDP, both the amide I and amide II bands were redshifted. It was shown that the interaction of the two changed the conformation of HEDP. The strength of the Myr-HEDP hydrogen bond band was higher and wider than that of HEDP, which indicated that the hydrogen bond between HEDP and Myr occurred. Hydrogen bonding may be caused by the interaction of glutamine residues in HEDP with hydroxyl groups in Myr [32].

To further expose the secondary structure changes of HEDP and Myr-HEDP, the FT-IR curves of HDEP, and Myr-HEDP in the range of 1600–1700 cm^−1^ were fitted. The amide I strip (1700–1600 cm^−1^) includes α-helix (1650–1660 cm^−1^), β-sheet (1660–1670 cm^−1^), β-turn (1670–1680 cm^−1^), and random coil (1680–1690 cm^−1^) [33]. As shown in Figure 7F, the secondary structure content of HEDP varied dramatically depending on the pH levels, and as the pH increased, the β-turn and β-sheet contents increased and reduced, respectively. This suggested that the HEDP’s secondary structure was impacted by pH in some way. Moreover, the secondary structure content of HDEP and Myr-HEDP was also different. The change was most pronounced at pH 9.0, and when combined with Myr, its random coil content decreased, and the β-sheet content increased from 18% to 38%. The β-sheet structure consists of parallel peptide chains or anti-parallel peptide chains connected by hydrogen bonds. This spatial conformation is more conducive to binding with flavonoids [34], so it can be seen that the Myr-HEDP formed under the condition of pH 9.0 was more stable. It should be noted that an increase in β-sheet content may also indicate stronger intermolecular association and reduced molecular flexibility [35,36]. However, considering the relatively small particle size, low PDI, and high absolute zeta potential of Myr-HEDP at pH 9.0, this conformational rearrangement is more likely associated with the formation of stable nanomicelles rather than uncontrolled aggregation. The more compact β-sheet-rich structure may strengthen the interaction between HEDP and Myr, thereby slowing Myr release during simulated digestion and contributing to improved digestive stability and delivery performance.

### 3.7. Digestion Characteristics

**Figure 8:**
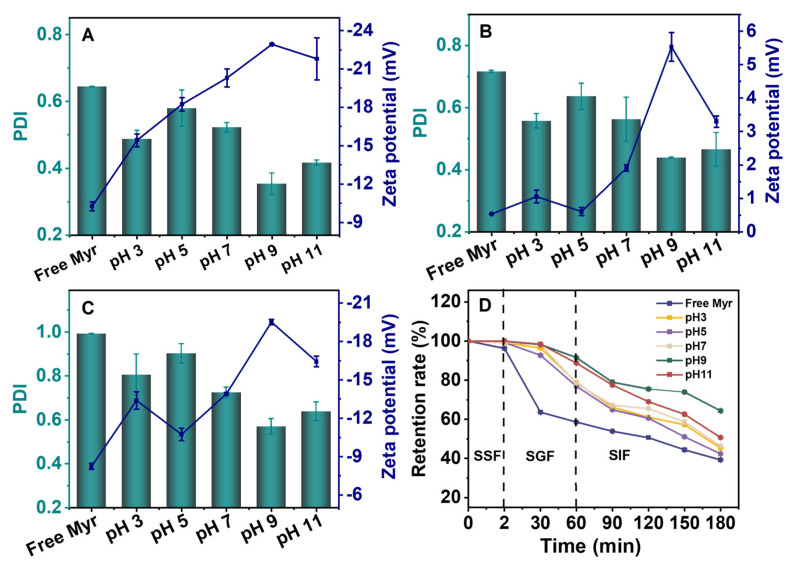
Simulated in vitro digestion of Myr-HEDP composite micelles at different pHs: zeta potential and PDI: (**A**) simulation of the oral environment; (**B**) simulation of the gastric environment; (**C**) simulation of the intestinal environment; (**D**) simulation of retention profiles under in vitro digestion.

When the delivery carrier passes through the digestive tract, it is difficult for the functional components to exert their true biological activity due to the pH and digestive enzymes. Studying the digestive characteristics of Myr-HEDP is the key index to evaluate its delivery effect. Figure 8 shows the variations in retention rate, particle size, PDI, and zeta potential of free Myr and self-assembled Myr-HEDP at different pH levels during the simulated digestion process. According to Figure 8A–C, the PDI values of all pH-modulated self-assembled Myr-HEDP increased after 2 h of incubation in SGF (pH 2.0), which could be attributed to the shearing effect of pepsin on HEDP in simulated gastric juice, which led to the disruption of the microstructure of HEDP, and then electrostatic deposition between Myr and HEDP, and finally, the Myr-HEDP composite nanomicelle aggregation. At the same time, both Myr-HEDP were positive and close to 0, which may be due to the pH of the simulated gastric fluid being 2.0, which is less than the isoelectric point of edible dock protein. As shown in Figure 8D, after 2 h of simulated intestinal digestion, the retention of free Myr was 58.60% and 39.26% under SIF conditions. The retention of Myr-HEDP at pH 9.0 in SGF conditions (91.54%) was much higher than that in SIF (64.31%). After HEDP encapsulation, Myr was released in small amounts in SGF and in large amounts in SIF, which is an important feature of intestinal-targeted delivery for effective absorption and utilization of functional factors. The particle sizes of Myr-HEDP in SIF were all decreased, and the absolute zeta potential and PDI were both significantly lower relative to the initial undigested Myr-HEDP, suggesting that the stability of the Myr-HEDP composite nanomicelle system was reduced and enzymatically cleaved by digestive enzymes in SIF, which is also conducive to the intestinal absorption of Myr. Thus, in summary, HEDP prevents Myr leakage in the stomach and confers Myr pH-triggered intestinal-targeted release properties to enhance the oral absorption of Myr.

### 3.8. UV, Thermal, and Storage Stability of Myr-HEDP Composite Nanomicelles

**Figure 9:**
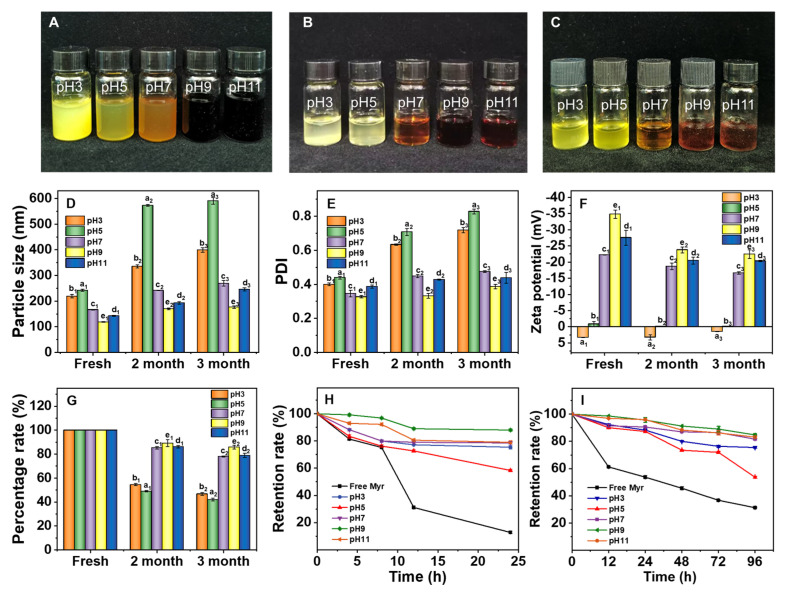
Storage stability, UV stability, and thermal stability of Myr-HEDP composite micelles at different pH values: (**A**) visualization of initial samples; (**B**) samples after 2 months of storage at 4 °C protected from light; (**C**) samples stored at 4 °C protected from light for 3 months; samples at 4 °C protected from light initially, and after 2 and 3 months of storage; (**D**) particle sizes; (**E**) PDI; (**F**) zeta potential; (**G**) retention rate; retention of samples (**H**) heated at 60 °C; (**I**) under UV irradiation. Different letters above the bars indicate statistically significant differences among samples (*p* < 0.05).

The variations in particle size, PDI, zeta potential, and Myr retention of self-assembled Myr-HEDP composite nanomicelles at different pHs were measured after storage at 4 °C for 3 months. The 3-month storage period at 4 °C was selected to evaluate the long-term stability of the Myr-HEDP nanomicelle system under refrigerated conditions commonly used for functional beverages, protein-based formulations, and nutraceutical food products. From Figure 9A–C, the visualization of the samples showed that Myr was gradually degraded with time, the solution became lighter in color, and unequal deposits were formed at the bottom of the test tubes, which indicated that some Myr-HEDP had aggregated and precipitated during storage. This phenomenon may occur through several mechanisms: (i) desorption of Myr from the HEDP surface; (ii) dissolution of the populin molecules from the interior of HEDP, followed by recrystallization; and (iii) reduced repulsive interactions between Myr-HEDP nanoclusters due to a decrease in the absolute value of the zeta potential. In addition, as shown in Figure 9D–F, the particle size, PDI, and zeta potential values of Myr-HEDP prepared under pH 9.0 conditions did not change significantly after 3 months of storage. The remaining four pH conditions exhibited a significant decrease in zeta potential and a considerable increase in particle size and PDI of the Myr-HEDP nanomicelles. It may be attributed to the decrease in electrostatic repulsion of the micellar particles during storage, leading to the aggregation of molecules, suggesting that the micellar system became unevenly dispersed after storage. In addition, the retention of Myr after 3 months of storage (Figure 9G) was as follows: pH 9.0 > pH 11.0 > pH 7.0 > pH 3.0 > pH 5.0. The results indicate that the binding capacity of HEDP to Myr was stronger under alkaline conditions, and the nanostructures formed by HEDP were denser, resulting in greater storage stability of the composite nanomicelles. The retention of Myr in the self-assembled HEDP under alkaline conditions was greater than 75% after 3 months of storage at 4 °C under light protection. These phenomena indicate that the self-assembled HEDP nanomicelles under alkaline conditions had a good protective effect on Myr and could significantly improve its storage stability. Although slight aggregation and precipitation were observed during prolonged storage, the HEDP nanomicelles prepared under alkaline conditions, particularly at pH 9.0, exhibited significantly improved physicochemical stability compared with those prepared under other pH conditions. The results suggest that HEDP nanomicelles are good delivery systems for hydrophobic nutrients and have potential applications in functional foods.

Myr is known to be unstable and is subject to degradation under the stimulus of light, heat treatment, and oxygen, leading to a decrease in its bioactivity and a significant decrease in bioavailability [1]. Therefore, the assessment of the stability of the delivery system under light and heat is important. In this study, the effects of different external factors (temperature and UV exposure time) on the stability of Myr-HEDP were investigated. With the increase in heating time (Figure 9H) and UV radiation (Figure 9G), the retention of free Myr gradually decreased to 12.74% and 31.21% at 24 h and 96 h, respectively. The retention of Myr in HEDP also decreased gradually, but the degradation rate was much lower than that of free Myr, in which Myr in the Myr-HEDP complex at pH 9.0 had the highest retention rates of 88.06% and 84.76% after heating treatment at 60 °C for 24 h and UV irradiation for 96 h, respectively. It is speculated that Myr in HEDP can improve the thermal and photostability of Myr through the hydrogen bonding effect, embedding Myr as a stable amorphous form in the HEDP matrix and inhibiting the escape of Myr molecules. Xiao et al. also reported that Cur-kafirin/carboxymethyl chitosan nanoparticles exhibited better photochemical protection attributed to the thicker physical barrier of the polymer matrix [27].

## 4. Conclusions

In this study, HEDP had the highest encapsulation efficiency and loading capacity for Myr at pH 9.0. Moreover, at this pH, the interaction affinity between HEDP and Myr was the strongest, driven mainly by hydrogen bonding, hydrophobic interactions, and van der Waals forces. Importantly, the Myr encapsulated in Myr-HEDP nanomicelles exhibited more stable UV, thermal, storage, and digestive stability than at other pH values. In summary, the present results demonstrated that HEDP exhibited favorable physicochemical characteristics, encapsulation performance, and stability enhancement effects for myricetin under the investigated conditions, indicating its potential as a plant protein-based delivery material for hydrophobic bioactive compounds.

## Figures and Tables

**Table 1:** Thermodynamic parameters of the binding interaction between edible dock protein and Myr compounds at different temperatures.

Sample	T (*K*)	*K_SV_* (10^3^ L·mol^−1^)	*K_q_* (10^11^ L·(mol·s)^−1^	*K_a_* (10^3^ L·mol^−1^)	n	*∆H* (KJ·mol^−1^)	*∆G* (KJ·mol^−1^)	*∆S* (J·(mol·K)^−1^)
	298	1.5	1.5	0.0166	1.2199	-	−11.96	-
pH 7	308	2.2	2.2	1.2437	1.1953	−37.8262	−11.58	−23.2343
	318	1.6	1.6	0.008	0.9047	-	−11.21	-
	298	1.1	1.1	4.1504	0.6181	-	−17.16	-
pH 9	308	1.9	1.9	5.8573	0.553	−20.1481	−3.91	−9.91361
	318	1.0	1.0	3.1937	0.7677	-	−3.97	-
	298	3.9	3.9	1.848	0.9237	-	−20.31	-
pH 11	308	3.5	3.5	5.583	0.9773	−21.7810	−20.53	−13.817
	318	1.8	1.8	1.2218	0.9445	-	−20.74	

## Data Availability

The original contributions presented in the study are included in the article; further inquiries can be directed to the corresponding author.
